# Annotations

**Published:** 1910-09-24

**Authors:** 


### Annotations.

### Spotted Fever.

From various parts of the country cases of epidemic cerebrospinal meningitis have been notified, and during the past week several new cases have been reported. The public has an unfortunate conception of the disease, and is inclined to regard it as one of the most dangerous visitants that can reach us. The notification of an individual case lately was sufficient to create a complete scare in a city where, as medical men know, the disease is not so uncommon as may be supposed—certainly by no means so rare as typhus. Although we do not in the least desire to contend that proper and necessary precautions should not be taken against an outbreak of the disease, we would yet venture to doubt the advisability of notifying sporadic cases and thereby creating an amount of alarm and consternation which is quite out of proportion to the real danger to the community. Cerebrospinal meningitis is probably as dangerous from a point of view of infectivity as typhoid, and where it occurs epidemically it is *ipso facto* evidence of insanitation and bad hygiene. But the presence of a solitary patient suffering from the disease is not sufficient to put a whole village or street into quarantine, and the necessity for strict isolation of "contacts" is much less urgent than, let us say, in cases of measles or mumps, and certainly not so urgent as in cases of whooping-cough! This is one of the points upon which the profession should reassure the public, which at present is scared out of its senses by the mere whisper of spotted fever. Fear of infection is a very wholesome prophylactic at times, but it may be carried to extremes, and the present scare is founded on a total misapprehension of the incidence and causation of the disease.

### The Scottish Medical Schools.

Professor Sims Woodhead, who has been a member of the Committee on the Scottish Universities, has embodied his observations on the clinical teaching in the medical schools in a note which is appended to the report of the committee. He starts with the explanation that as a result of the high reputation of the Edinburgh and Glasgow Schools a very large number of students have flocked to them to participate in the training given there. As a result there has been some difficulty in providing sufficient clinical material on which to base the instruction of students in the later years of the curriculum. At Glasgow recent rearrangements have solved this problem; but in Edinburgh, notwithstanding some reforms which have been attended by considerable success, the difficulty is still great. Professor Woodhead says that much more might be done in the utilisation not only of in-patients but also of out-patients, for clinical instruction. He points out that, though the rivalry between the University teachers and those of the Extra-Mural School is beneficial by encouraging independence and competition, yet "the success of any competitive element amongst these teachers can only result in the unequal distribution of students amongst the teachers, and, therefore, of the available clinical material amongst the students." Some modification of this system is now desirable if the reputation of the school is to be raised or even maintained. How important it is to make the most of every possible clinical facility can be understood from Professor Woodhead's calculation that there are about 200 students of surgery entering the wards each year, and that available beds (surgical) amount to but 336. Then, too, the professors of systematic and clinical surgery and medicine are greatly overworked, both as regards clinical teaching and examinations. The remedy proposed is, roughly, to put much more teaching responsibility on the assistant physicians and assistant surgeons; it would then be possible to divide the clinical students into senior and junior classes. This would also lead to much more utilisation of out-patient clinical material, and to more practical ward teaching, because a much larger proportion of the class would be able to examine each case. It involves, practically, an approach towards the London system as against the Edinburgh tradition. Whether it is practical, as a matter of politics, is a matter for local decision; but it is clear that something must be done, and there seems no inherent reason why a system which works smoothly in England should not do so also in Scotland.

### Farabœuf.

Surgery, and in particular that of the French school, owes a debt of gratitude to the eminent anatomist and surgeon Farabœuf, who passed away on August 14 last. Born at Bannost on May 6, 1841, of parents in humble circumstances, he was educated at the College of Provins, entering the Faculty of Medicine of Paris in 1859 and becoming hospital interne in 1864. He graduated M.D. in 1871, was elected Prosector in Anatomy in 1872 and Fellow of the Faculty of Medicine in 1875. He became Director of the Anatomical School in 1878. At his death he was a member of the Academy of Medicine, honorary Professor of the Paris Faculty of Medicine, and officer of the Legion of Honour. He published many works on anatomical and surgical subjects, some of which are standard textbooks in France to-day. From the eloquent tribute paid to his memory by his friend and former pupil, Professor Delbet, we gather that Farabœuf was endowed with an extraordinary gift of teaching. Rough in his manner and apparently intolerant of mediocrity, his ascetic exterior hid a kindly heart. He somewhat resembled the old anchorites of Thebes in that he seemed to be persuaded that the only expression of affection allowable consisted in the infliction of chastisement. His teaching was extremely thorough, and such of his pupils as were not driven away in disgust profited greatly by their association with him. He did not regret the absentees, for he wished to have in the profession those only whose hearts were in their work. By his death yet another link with the famous past is snapped.

